# Imperatorin inhibits mitogen‐activated protein kinase and nuclear factor kappa‐B signaling pathways and alleviates neuroinflammation in ischemic stroke

**DOI:** 10.1111/cns.13748

**Published:** 2021-10-21

**Authors:** Jian‐wei Ge, Shi‐ji Deng, Zhi‐wei Xue, Pin‐yi Liu, Lin‐jie Yu, Jiang‐nan Li, Sheng‐nan Xia, Yue Gu, Xin‐yu Bao, Zhen Lan, Yun Xu, Xiao‐lei Zhu

**Affiliations:** ^1^ Department of Neurology Drum Tower Hospital Medical School and The State Key Laboratory of Pharmaceutical Biotechnology Nanjing University Nanjing Jiangsu PR China; ^2^ Institute of Brain Sciences Nanjing University Nanjing Jiangsu PR China; ^3^ Jiangsu Key Laboratory for Molecular Medicine Medical School of Nanjing University Nanjing Jiangsu PR China; ^4^ Jiangsu Province Stroke Center for Diagnosis and Therapy Nanjing Jiangsu PR China; ^5^ Nanjing Neuropsychiatry Clinic Medical Center Nanjing Jiangsu PR China; ^6^ Department of Neurology Drum Tower Hospital of Nanjing Medical University Nanjing Jiangsu PR China

**Keywords:** imperatorin, ischemic stroke, MAPK pathway, microglia, neuroinflammation, NF‐κB pathway

## Abstract

**Aims:**

Microglia‐mediated neuroinflammation plays an important role in the pathological process of ischemic stroke, and the effect of imperatorin on post‐stroke neuroinflammation is not fully understood.

**Methods:**

Primary microglia were treated with imperatorin for 2 h followed by LPS (100 ng/ml) for 24 h. The expression of inflammatory cytokines was detected by RT‐PCR, ELISA, and Western blot. The activation of MAPK and NF‐κB signaling pathways were analyzed by Western blot. The ischemic insult was determined using a transient middle cerebral artery occlusion (tMCAO) model in C57BL/6J mice. Behavior tests were used to assess the neurological deficits of MCAO mice. TTC staining was applied to measure infract volume.

**Results:**

Imperatorin suppressed LPS‐induced activation of microglia and pro‐inflammatory cytokines release and attenuated ischemic injury in MCAO mice. The results of transcriptome sequencing and Western blot revealed that downregulation of MAPK and NF‐κB pathways might contribute to the protective effects of imperatorin.

**Conclusions:**

Imperatorin downregulated MAPK and NF‐κB signaling pathways and exerted anti‐inflammatory effects in ischemic stroke, which indicated that imperatorin might be a potential compound for the treatment of stroke.

## Graphical abstract

Imperatorin inhibits MAPKs and NF‐κB signaling pathways and alleviates neuroinflammation in ischemic stroke.

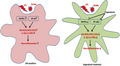

## 1 INTRODUCTION

Ischemic stroke is one of the most common causes of death and disability in the world.^1^ However, the pathological mechanisms of ischemic brain injury are not fully understood. Increasing evidence indicates that neuroinflammation plays an important role in ischemic stroke.^2^

Microglia, the resident macrophage in central nervous system (CNS), contributes to neuroinflammation after CNS injury.^3^, ^4^ After being activated by various stimuli, microglia upregulates several pro‐inflammatory mediators, including interleukin‐1β (IL‐1β), IL‐6, tumor necrosis factor‐α (TNF‐α), nitric oxide (NO), and prostaglandin E2 (PGE2).^5^ Interestingly, microglia activation exerts both protective and detrimental effects on ischemic stroke.^6^ Microglia activation increases the release of several neurotoxic cytokines.^7^ On the other hand, several studies have shown that microglia, the dynamic scavengers of cellular debris, contribute to the CNS homeostasis after ischemic stroke.^8^ Thus, modulation of microglia function is an attractive therapeutic strategy for the treatment of ischemic stroke.

Imperatorin (IMP), a linear furanocoumarin with molecular weight 270.28, is extracted from *Angelica sinensis*, *Angelica dahurica*. Traditional Chinese Medicine Database and Analysis Platform (TCMSP) has shown that IMP has high oral bioavailability and great blood‐brain barrier permeability.^9^ IMP has shown potential biological activities in various disease models, such as anti‐inflammatory,^10^ anti‐tumoral,^11^ and anti‐oxidative effects.^12^ IMP activates Nrf2‐mediated anti‐oxidative pathway to protect the RAW264.7 cells from inflammatory damage.^12^ IMP ameliorates lipopolysaccharide (LPS)‐induced memory deficit and attributes to the suppression of pro‐inflammatory response.^13^ In addition, IMP attenuates neuronal apoptosis and improves synaptic plasticity in a vascular dementia model.^14^ In this study, we explored the effects of IMP on the activation of microglia and neuroinflammation in ischemic stroke, which might provide an alternative strategy for the treatment of ischemic stroke.

## 2 MATERIALS AND METHODS

### 2.1 Reagents

IMP (CAS: 482‐44‐0, purity: ≥98%) was purchased from ALADDIN Ltd. (Shanghai, China) and dissolved in 0.1% dimethyl sulfoxide (DMSO) for the follow‐up cell experiments. As for *in vivo* experiment, IMP was dissolved in 0.9% saline with 5% DMSO. LPS (Escherichia coli 055: B5) was obtained from Sigma‐Aldrich (St. Louis, MO, USA).

### 2.2 Primary microglia culture

Primary microglial cells were isolated from newborn C57BL/6J mice as previously described.^15^ Cells were cultured in DMEM (Invitrogen) supplemented with 10% fetal bovine serum (FBS, Hyclone) in a 37°C humidified incubator for 10 days. Then, the separated microglial cells were seeded into 12‐ or 24‐well plates for the subsequent experiments. The purity of the primary microglia was greater than 95%, which was examined by immunocytochemistry analysis using Iba‐1 antibody (1:500; Wako, Japan).

### 2.3 Animals and middle cerebral artery occlusion (MCAO)

All animal experiments were approved by the Animal Care and Use Committee at Nanjing University and designed according to the ARRIVE guidelines 2.0.^16^ Male C57BL/6J mice (6‐ to 8‐week‐old) weighing 22 ± 2 g were used in this study. Mice were housed in a facility at a light cycle of 12‐hr light/12‐hr dark, with access to water and food *ad libitum*. Sixty mice were randomly divided into three groups, including sham‐operate (SHAM) group, 5% dimethyl sulfoxide‐treated MCAO (MCAO + DMSO) group, and IMP (5 mg/kg)‐treated MCAO (MCAO + IMP) group. Transient MCAO model was prepared as previously described.^17^ Briefly, mice were anesthetized and maintained at 37±0.5°C on a heating pad. Under the dissecting microscope, the right common carotid artery and external carotid artery (ECA) were isolated. A piece of 6/0 monofilament nylon suture with a heat‐rounded tip (Doccol corporation, MA, USA) was inserted into ECA and arranged to obstruct the origin of MCA. Laser Doppler flowmetry (Perimed corporation, Stockholm, Sweden) was used to assess the cerebral blood flow. Mice were subjected to 60 min of occlusion, and the monofilament was withdrawn to allow blood reperfusion. All the mice were included in this study when the laser Doppler reading was below 30% of baseline and no hemorrhage occurred. Sham‐operated animals underwent the same procedure without monofilament nylon suture inserting. The mice were treated with IMP (5 mg/kg) or vehicle intraperitoneally at 30 min, 24 h, and 48 h post‐reperfusion.

### 2.4 Measurement of infarct volume

Infarct volume was measured by 2, 3, 5‐triphenyltetrazolium chloride (TTC, Sigma‐Aldrich) staining as previously described.^18^ Briefly, brains were cut into 1‐mm‐thick coronal slices and immersed in 2% TTC at 37°C. The photographs of the sections were taken with a digital camera and analyzed by ImageJ (ImageJ 1.5, NIH). The percentage of infarct was calculated as [(VC − VL)/VC] × 100, where VC was the volume of the infarcted tissue in the contralateral hemisphere and VL was the volume of the non‐infarcted tissue in the ipsilateral hemisphere.

### 2.5 Neurobehavioral tests

Modified neurological severity scores (mNSS) test was used for neurological function assessment at 24 and 72 h after perfusion as previously described.^19^ The forelimb grip strength experiment was used to assess the motor function of the mice using a grip strength meter (GS3, Bioseb). During the experiment, mice were performed to hold a grid which was connected to an electronic digital force gauge. All mice were then gently pulled backward from the base of the tail and the maximum value was recorded. The data were averaged from five measurements of each mouse.

The rotarod experiment was performed to assess sensorimotor coordination and balance.^20^ Briefly, mice were trained in the rotaroad (RWD Life Science) for 3 days before MCAO. During the test, the rotating rod was accelerated from 4 to 40 rpm in 5 min. All tests were performed twice with a 5‐min period and a 10‐min interval. The time when mice fell from the rotating rod was recorded. All neurobehavioral tests were performed by an experimenter who was blind to the experimental group.

### 2.6 LPS‐treated microglia inflammation model

Primary microglia were divided into 3 groups as follows: control group, LPS (100 ng/ml) treatment group, and IMP treatment group. In the IMP‐treated group, microglia were incubated with IMP (10, 30, or 50 μM) for 2 h, and then, LPS was added for another 24 h.

### 2.7 Nitrite analysis

The concentration of nitrite in the supernatant produced by microglia was detected with a Griess reaction, which had high sensitivity and specificity for nitrite and nitrate by online reduction of nitrate to nitrite by cadmium and reduced copper, according to the manufacturer's instructions (Beyotime Biotech Nantong). Briefly, primary microglia were pretreated with IMP for 2 h and treated with LPS (100 ng/ml) for 24 h. Then, the culture media were collected, and the OD was measured at 540 nm.

### 2.8 Enzyme‐linked immunosorbent assay (ELISA)

The levels of cytokines including PGE2, IL‐1β, IL‐6, and TNF‐α in the supernatant were measured using the commercial ELISA kits as described in the manufacturer's protocol (Cusabio Biotech). The expression of the cytokines was obtained with a standard curve.

### 2.9 Flow cytometry

Primary microglia were digested, washed with phosphate‐buffered saline (PBS) twice, and re‐suspended in cold PBS at a density of 1 × 10^6^ cells/ml. The microglia were incubated with CD86‐APC or Arg1‐APC in dark at 4°C for 30 min, and the expression of CD86 or Arg1 was detected by a FACS Calibur flow cytometer (BD Biosciences).

### 2.10 Reverse transcription and quantitative real‐time PCR

TRIzol reagent (Invitrogen Life Technologies, Carlsbad, CA, USA) was used to extract total RNA as described in our previous study.^21^ Briefly, 1000 ng of total RNA was reverse transcribed in a 20 μl reaction at 37°C for 15 min followed by 85°C for 5 s using PrimeScript RT Master Mix (Vazyme Biotech Co.,ltd). Real‐time PCR was performed on a Step One Plus PCR system (Applied Biosystems) using a SYBR Green Kit (Applied Biosystems).

The primers were as follows:

iNOS Forward: CAGCTGGGCTGTACAAACCTT,
Reverse: CATTGGAAGTGAAGCGTTTCG;
COX‐2 forward: TCTCCAACCTCTCCTACTAC,
Reverse: GCACGTAGTCTTCGATCACT;
IL‐1β Forward: AAGCCTCGTGCTGTCGGACC,
Reverse: TGAGGCCCAAGGCCACAGGT;
IL‐6 Forward: GCTGGTGACAACCACGGCCT,
Reverse: AGCCTCCGACTTGTGAAGTGGT;
TNF‐α Forward: CAAGGGACAAGGCTGCCCCG,
Reverse: GCAGGGGCTCTTGACGGCAG;
CD86 Forward: TGTTTCCGTGGAGACGCAAG
Reverse: TTGAGCCTTTGTAAATGGGCA
Arg1 Forward: CTCCAAGCCAAAGTCCTTAGAG
Reverse: AGGAGCTGTCATTAGGGACATC
GAPDH Forward: GCCAAGGCTGTGGGCAAGGT,
Reverse: TCTCCAGGCGGCACGTCAGA.

### 2.11 Western blot

The protein of brain tissues and microglia were extracted and quantified as previously described.^22^, ^23^ Equal quantities of proteins were separated by 10% SDS‐PAGE and then transferred to PVDF membranes. The membranes were blocked with 5% skim milk for 2 h at room temperature, and then, the membranes were incubated with primary antibodies against iNOS, IL‐6, IL‐1β, TNFα, ERK1/2, p‐ERK1/2, JNK, p‐JNK, p38, p‐p38, NF‐κBp65, p‐NF‐κBp65 (1:1000, Cell Signaling Technology), COX‐2, or β‐actin (1:5000, Bioworld Biotechnology) overnight at 4°C, and then, the membranes were incubated with HRP‐conjugated secondary antibodies (Cell Signaling Technology) for 1 h at room temperature. The protein bands were visualized with the ECL Detection Kit (Bioworld Biotechnology). Images were acquired using the Gel‐Pro system (Tanon Technologies), and the intensity of each band was analyzed using ImageJ software (ImageJ 1.5, NIH).

### 2.12 Immunofluorescence staining

Mice brains were fixed with 4% paraformaldehyde and cut into 20‐μm sections using a Leica CM1900 cryostat after the gradient of dehydration. Microglia cells were fixed with 4% paraformaldehyde for 15 min. The brain sections and cell coverslips were washed 3 times with PBS, blocked in 2% fetal bovine serum, and incubated overnight at 4°C with following primary antibodies: Iba1 (1:500; Wako, Japan) or NF‐κBp65 (1:200; Cell Signaling Technology). Then, these samples were incubated with indicated secondary antibodies for 2 h in dark at room temperature. DAPI (10 μg/ml) was added for 15 min to stain nuclei. The images were acquired with an Olympus BX51 (Japan) fluorescence microscope, and the relative area and integrated optical density (IOD) of microglia were analyzed using ImageJ software (ImageJ 1.5, NIH).

### 2.13 Transcriptome sequencing

Primary microglia were treated with 50 μM of IMP or DMSO for 2 h followed by stimulation with LPS (100 ng/ml) for 24 h. Total RNA was extracted with TRIzol (Invitrogen, USA) from the treated microglia. Sequencing was performed on an Illumina Hiseq 2000 platform by Shanghai Majorbio Bio‐pharm Technology Co., Ltd. The differentially expressed genes (DEGs) were filtered with a *p* ≤ 0.05 and fold change ≥2 and subjected to Kyoto Encyclopedia of Genes and Genomes (KEGG).

### 2.14 Statistical analysis

All data were expressed as the mean ± standard deviation (SEM) of at least 3 independent experiments and analyzed by SPSS 20.0 (SPSS 20.0 software. USA). Shapiro‐Wilk test was used to test the normality assumption of the data. Student's t test was used to compare differences between two groups if the data are normal distribution, while Mann‐Whitney test was applied to compare the non‐normally distributed variables. For more than two groups, statistical difference was analyzed by one‐way analysis of variance (ANOVA) followed by Bonferroni's post hoc test or by the Kruskal–Wallis test followed by Dunn's multiple comparison test. *p* < 0.05 was considered to be statistically significant.

## 3 RESULTS

### 3.1 IMP suppresses LPS‐induced inflammatory cytokines expression in primary microglia

**FIGURE 1:**
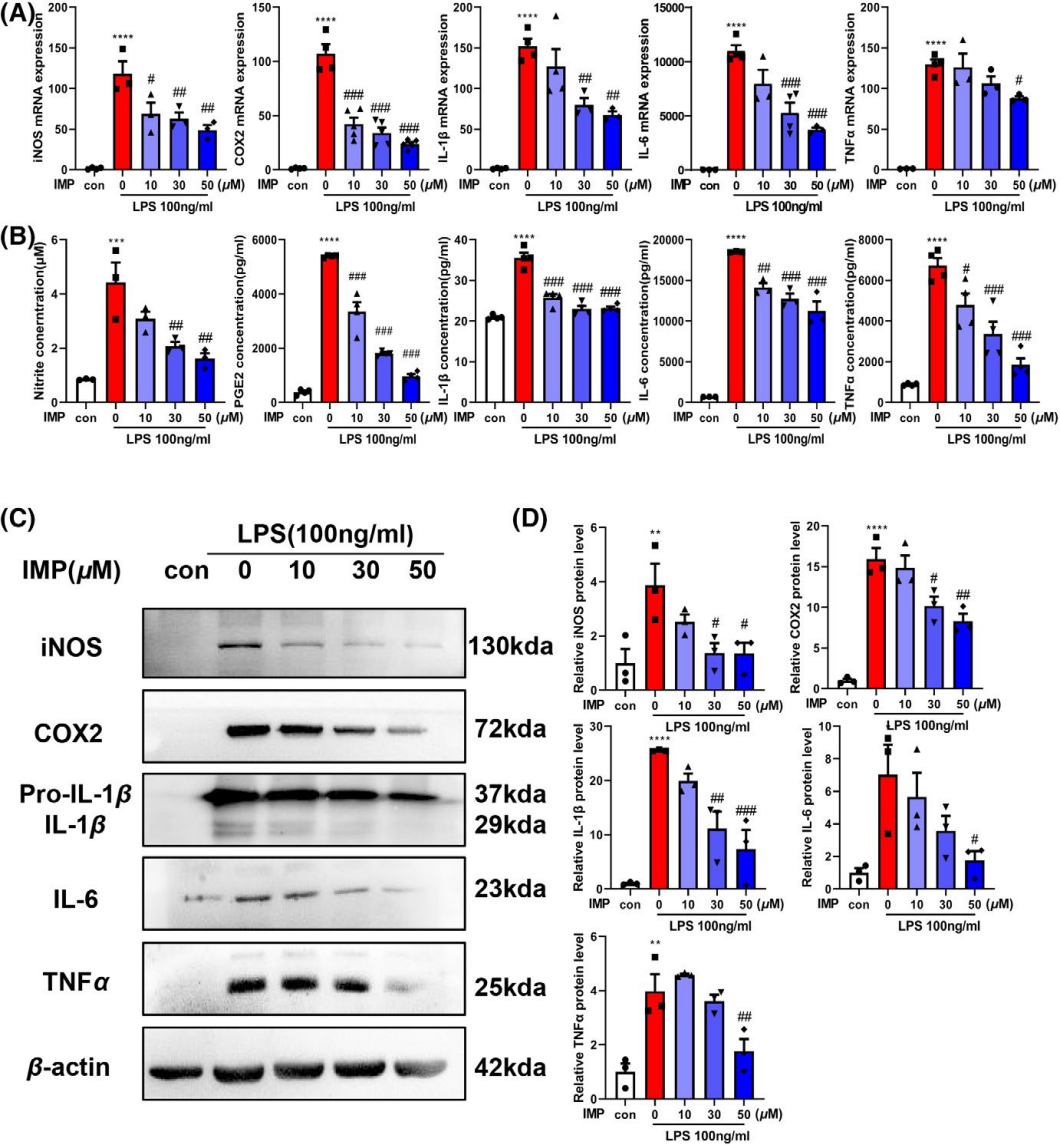
IMP decreased the production of LPS‐induced inflammatory cytokines expression in primary microglia. Microglia was pretreated with various concentrations of IMP (10, 30, 50 μM) for 2 h followed by LPS treatment (100 ng/ml). (A) The mRNA levels of iNOS, COX2, IL‐1β, IL‐6, and TNFα were measured using real‐time PCR after LPS treatment for 24 h with or without IMP. (B) The concentrations of NO, PGE2, IL‐1β, IL‐6, and TNFα in the supernatants of primary microglia were detected using the Griess reaction and ELISAs. (C, D) The protein levels of iNOS, COX2, IL‐1β, IL‐6, and TNFα were analyzed via Western blot β‐actin as an internal reference. The grey scale values of iNOS, COX2, IL‐1β, IL‐6, and TNFα in the blots were quantified by ImageJ software and normalized to β‐actin and were represented as the fold change. The values were presented as the means ± SEM. The data shown here were representative of three independent experiments. *p < 0.05, **p < 0.01, ***p < 0.001, ****p < 0.0001 vs. control group; #*p* < 0.05, ##*p* < 0.01, ###*p* < 0.001vs. LPS‐treated group [Colour figure can be viewed at wileyonlinelibrary.com]

To evaluate the effects of IMP on neuroinflammation, the expression levels of inflammatory cytokines were examined in LPS‐treated primary microglia. LPS treatment significantly increased the mRNA levels of iNOS, COX2, IL‐1β, IL‐6, and TNFα, while IMP could partially rescue these effects (Figure 1A). In addition, IMP inhibited the production of NO, PGE2, IL‐1β, IL‐6, and TNFα in the supernatant (Figure 1B) and decreased the protein levels of iNOS, COX2, IL‐1β, IL‐6, and TNFα (Figure 1C,D) in LPS‐treated microglia. These results indicated that IMP suppressed LPS‐induced inflammatory cytokines expression in primary microglia.

### 3.2 IMP reverses LPS‐induced microglia morphological changes and decreases the activation of pro‐inflammatory microglia

**FIGURE 2:**
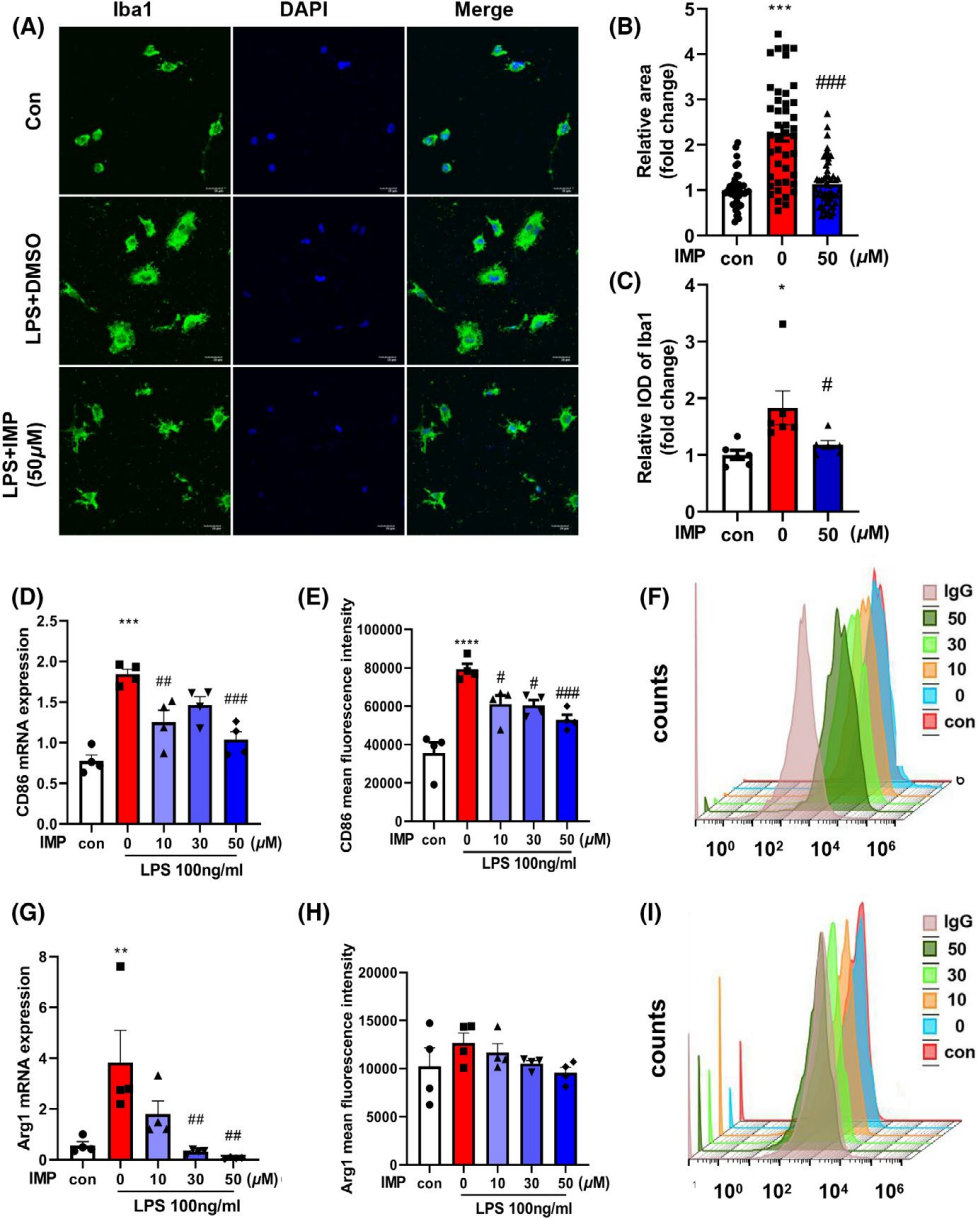
IMP reversed LPS‐induced microglia morphological changes and decreased the activation of pro‐inflammatory microglia. (A) Primary microglia were pretreated with different concentrations of IMP for 2 h and then treated with LPS for 24 h. The morphological feature was examined by immunocytochemistry analysis using Iba‐1 antibody. Scale bar = 20 μm. (B) Surface area of cells and (C) Integrated optical density (IOD) of Iba1 in (A) were measured by Image J. (D) The mRNA level of CD86 was measured using real‐time PCR. (E, F) Effects of IMP on the mean fluorescence intensity of CD86 measured using flow cytometry. (G) The mRNA level of Arg1 was measured using real‐time PCR. (H, I) Effects of IMP on the mean fluorescence intensity of Arg1 measured using flow cytometry. The values were presented as the means ± SEM. The data were representative of four independent experiments. *p < 0.05, **p < 0.01, ***p < 0.001, ****p < 0.0001 vs. control group; #*p* < 0.05, ##*p* < 0.01, ###*p* < 0.001vs. LPS‐treated group [Colour figure can be viewed at wileyonlinelibrary.com]

Since the expression of pro‐inflammatory cytokines was decreased by IMP treatment, we examined the effects of IMP on morphological feature of microglia. The results showed LPS treatment increased the size of soma and enhanced intensity of Iba1 fluorescence, which was inhibited by IMP pretreatment (Figure 2A–C). Comparing to control group, LPS treatment significantly increased the mRNA levels of CD86 and the amount of CD86^+^ microglia, and IMP pretreatment suppressed these effects (Figure 2D–F). Notably, the mRNA of Arg1, a marker of anti‐inflammatory microglia, was also decreased following IMP pretreatment. However, the amount of LPS‐induced Arg1^+^ microglia was not significantly changed following IMP pretreatment (Figure 2G–I). These results indicated that IMP reversed LPS‐induced microglia morphological changes and decreased the activation of pro‐inflammatory microglia.

### 3.3 IMP inhibits MAPK and NF‐κB signaling pathways in LPS‐induced microglia

**FIGURE 3:**
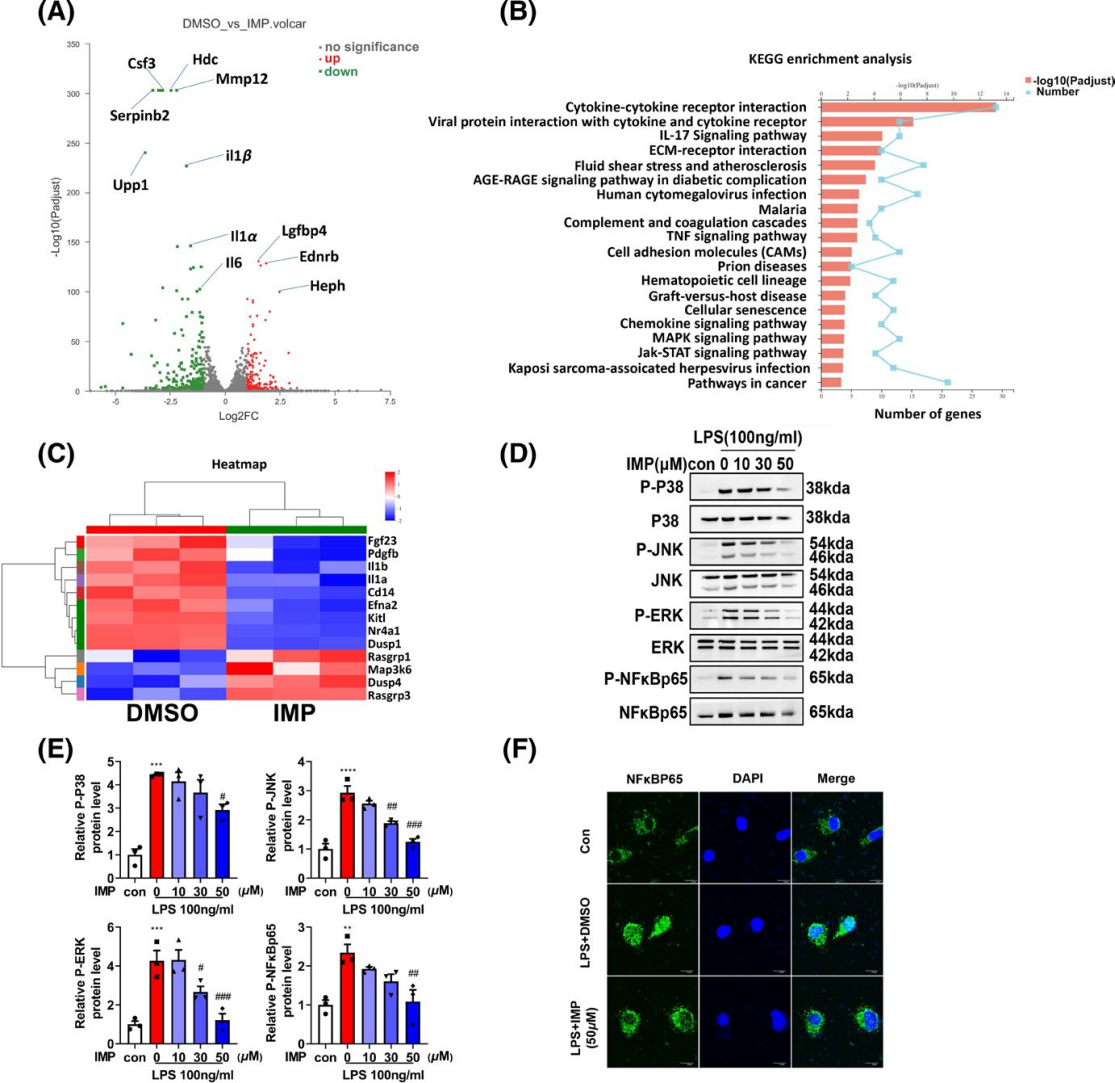
IMP decreased LPS‐induced activation of MAPK and NF‐κB pathways in primary microglia. Effects of IMP on the LPS‐induced microglia pathways were studied by RNAseq. (A) Volcano plot for microglia cells challenged with IMP + LPS versus DMSO + LPS group, the number of differentially expressed genes (DEGs) were listed. (B) Functional enrichment analysis of the KEGG pathway. (C) Heat map representing gene expression changes seen in IMP + LPS versus DMSO + LPS group in MAPK signaling pathway. n = 3 per group. Microglia were pretreated with various concentrations of IMP (10, 30, 50 μM) for 15 min followed by LPS treatment (100 ng/ml). (D, E) The protein expressions of p‐p38/p38, p‐JNK/JNK, p‐ERK/ERK, p‐ NF‐κBp65/NF‐κBp65 were analyzed by Western blot. The quantification of relative band intensities was determined by densitometry. (F) Microglia were treated with IMP and examined by immunocytochemistry analysis using NF‐κBp65 antibody. Scale bar = 10 μm. The values were presented as the means ± SEM. The data shown here were representative of three independent experiments. **p < 0.01, ***p < 0.001, ****p < 0.0001 vs. control group; #*p* < 0.05, ##*p* < 0.01, ###*p* < 0.001 vs. LPS‐treated group [Colour figure can be viewed at wileyonlinelibrary.com]

To further explore the mechanisms underlying the anti‐inflammatory effects of IMP in microglia during ischemic stroke, transcriptomic sequencing was performed and the data showed that 198 genes were downregulated and 145 genes were upregulated in IMP group (Figure 3A). KEGG pathway analysis showed that several pathways including IL‐17 signaling pathway, TNF signaling pathway, and mitogen‐activated protein kinase (MAPK) signaling pathways were involved (Figure 3B,C). Since MAPK pathways played a critical role in the neuroinflammation, we tested whether IMP affected the MAPK pathways in LPS‐induced microglia. The phosphorylation of JNK, ERK, and p38 was significantly increased by LPS treatment and decreased following IMP pretreatment (Figure 3D,E). NF‐κB pathway plays a critical role in inflammation, and it has been reported that NF‐κB pathway might act downstream of the MAPK signaling pathways.^24^ Here, we found that IMP decreased the phosphorylation of NF‐κBp65 in LPS‐treated microglia (Figure 3D,E) and interfered the translocation of NF‐κBp65 from cytoplasm to nucleus (Figure 3F). Overall, our data revealed that IMP inhibited MAPK and NF‐κB signaling pathways in LPS‐induced microglia.

### 3.4 IMP attenuates ischemic brain injury in MCAO mice

**FIGURE 4:**
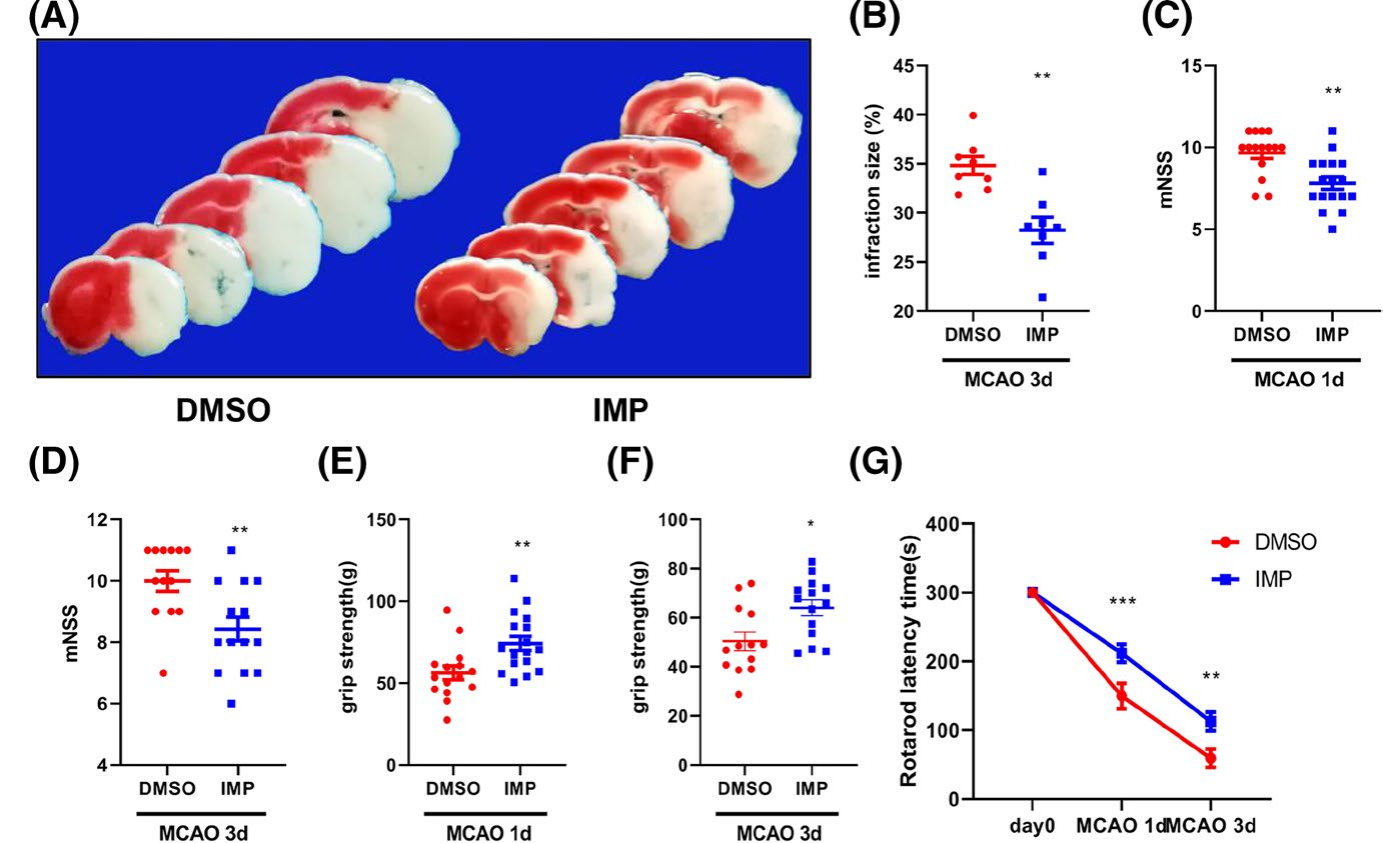
IMP decreased the infarct volume and improved neurological deficits after ischemic stroke. (A) Representative TTC staining and infarct volumes at 3 days after MCAO. (B) Infarct volume was determined in MCAO mice after IMP treatment (n = 8). The results of mNSS scores (C, D), grip strength test (E, F), and rotarod test (G) were examined in MCAO mice after IMP treatment. n=15–17 per group at MCAO 1 d, n = 13–14 per group at MCAO 3 d. The values were presented as the means ± SEM. *p < 0.05, **p < 0.01, ***p < 0.001vs. DMSO group [Colour figure can be viewed at wileyonlinelibrary.com]

Next, we investigated whether IMP could reduce brain damage and improve neurological deficits in ischemic stroke. The results of TTC showed that the infarct volume of MCAO mice with IMP treatment was significantly decreased as compared with that of DMSO groups (28.32% ± 1.31% in IMP group vs. 34.82% ± 0.92% in DMSO group, Figure 4A,B). Consistently, IMP treatment significantly alleviated the mNSS scores (7.82 ± 0.38 points in IMP group vs. 9.67 ± 0.35 points in DMSO group at 1 d, 8.43 ± 0.39 points in IMP group vs. 10.00 ± 0.34 points in DMSO group at 3 d, Figure 4C,D), improved grip strength (74.24 ± 4.30 g in IMP group vs. 56.26 ± 4.25 g in DMSO group at 1 d, 64.04 ± 3.27 g in IMP group vs. 50.43 ± 3.79 g in DMSO group at 3 d, Figure 4E,F) and motor function (211.80 ± 12.91 s in IMP group vs. 149.70 ± 18.41 s in DMSO group at 1 d, 112.80 ± 13.46 s in IMP group vs. 59.62 ± 13.29 s in DMSO group at 3 d, Figure 4G) after MCAO. Collectively, these data indicated that IMP attenuated ischemic brain injury and neurological deficits in the ischemic stroke mice.

### 3.5 IMP suppresses the production of inflammatory cytokines and microglial activation after ischemic stroke.

**FIGURE 5:**
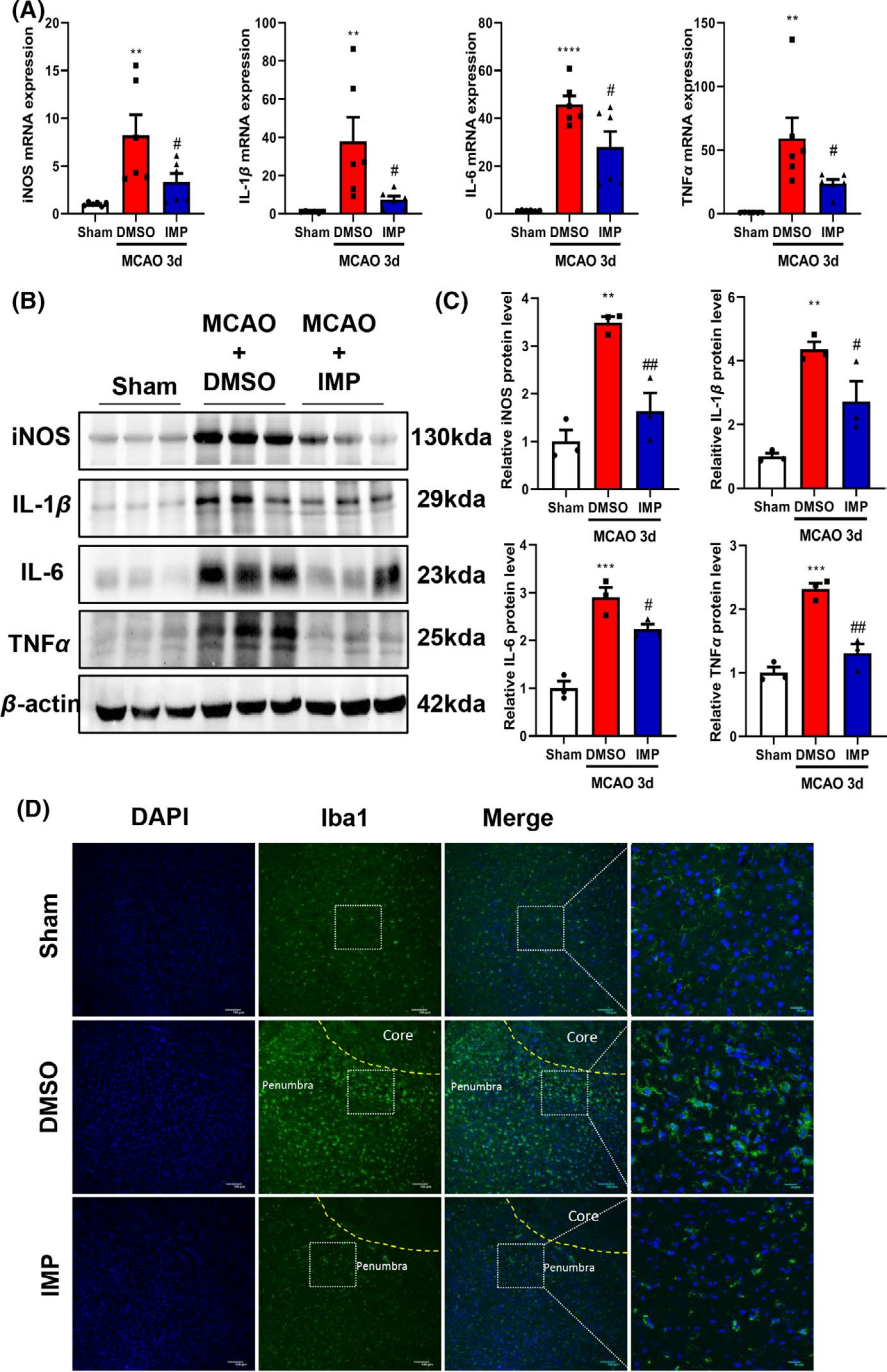
IMP suppressed the production of inflammatory cytokines and microglial activation in the brains of MCAO mice. (A) The mRNA levels of iNOS, IL‐1β, IL‐6, and TNF‐α in ischemic tissue were assessed by real‐time PCR. (B, C) The protein levels of iNOS, IL‐1β, IL‐6, and TNF‐α in ischemic tissue were assessed by Western blot. (D) Representative images of tissue sections from the ischemic penumbra collected 3 d after MCAO and stained with Iba1 and DAPI. Scale bar = 10 μm. n= 6 per group. **p < 0.01, ***p < 0.001, ****p < 0.0001 vs. sham group; #*p* < 0.05, ##*p* < 0.01 vs. DMSO group [Colour figure can be viewed at wileyonlinelibrary.com]

To determine the effects of IMP on neuroinflammation and microglia activation in MCAO mice, the mRNA levels of iNOS, IL‐1β, IL‐6, and TNF‐α were examined and it was shown that these pro‐inflammatory cytokines were reduced in the ischemic hemisphere of IMP groups (Figure 5A). Meanwhile, IMP treatment reversed MCAO‐induced increase of iNOS, IL‐1β, IL‐6, and TNF‐α protein levels (Figure 5B,C). In addition, the activation of microglia was significantly suppressed by IMP administration as demonstrated by the immunofluorescent staining of Iba‐1 (Figure 5D). Thus, our data indicated that IMP inhibited microglial activation and inflammatory response in the ischemic penumbra.

## 4 DISCUSSION

Microglia‐mediated neuroinflammation plays a critical role in the pathological mechanisms in ischemic stroke.^25^, ^26^ Here, we found that IMP, with good blood‐brain barrier (BBB) permeability,^27^ inhibited LPS‐induced microglial activation and reduced the expression of pro‐inflammatory cytokines, including iNOS, COX2, IL‐1β, IL‐6, and TNF‐α. Furthermore, IMP decreased the infarct volume and improved neurological deficits at 24 and 72 h after ischemic stroke and suppressed the microglial activation *in vivo*. Mechanistically, IMP restrained the activation of MAPK and NF‐κB signaling pathways in LPS‐induced microglia, which might be an effective therapeutic strategy for relieving the ischemic brain injury. Notably, several chemicals with similar structures of IMP have also shown anti‐inflammatory effects. For example, 8‐MOP (8‐methoxypsoralen) protects blood‐brain barrier via the Nrf‐2/HO‐1 pathway in experimental ischemic stroke.^28^ Bergapten ameliorates peripheral neuropathy induced by vincristine via inhibition of NF‐kappaB signaling and inflammatory cytokines.^29^ Isoimperatorin suppressed airway inflammation in the model of asthma.^30^ Therefore, further investigations into the structure analysis will be needed.

Microglia plays an important role in defending against pathogens, which maintains the homeostasis and inflammatory response.^31^, ^32^ However, excessive and persistent inflammatory response mediated by over‐activated microglia will release a large number of inflammatory mediators and neurotoxic substances.^33^ Overloaded IL‐1β, IL‐6, and TNF‐α act on adjacent microglia to induce neuroinflammation and glia‐activated feedback loops which aggravates the production of neurotoxic molecules.^34^ In addition, excessive NO inhibits mitochondrial cytochrome oxidase, which results in neuronal apoptosis.^35^ Minocycline treatment reduces infarct volume, hemorrhagic transformation, and blood‐brain barrier permeability after MCAO by inhibiting microglial activation.^36^ In addition, lncRNA‐1810034E14Rik attenuates brain injury by reducing microglia activation in experimental ischemic stroke.^37^ In the present study, our results showed IMP could suppress the microglial activation and reduce the expression of pro‐inflammatory cytokines. Notably, only male mice were used in this study. Since sex differences are found in the pharmacokinetic profiles of imperatorin and its 2 metabolites,^38^ and microglial functions vary dynamically between sex differences after stroke,^39^, ^40^ there might be sex differences in the protective effects of IMP against ischemic stroke, which will be explored in the following studies.

MAPK pathway is a major signal regulatory pathway sensitive to external stimuli.^41^ After the activation of microglia, MAPK pathways can be activated to release a large number of pro‐inflammatory cytokines and reactive oxygen species, leading to neuronal damage.^42^ Semaglutide, a kind of diabetes drug, reduces infarct size and inflammation and normalizes neurogenesis by suppressing the phosphorylation of p38 and ERK of MAPK signaling pathways in a rat model of stroke.^43^ Melatonin attenuates brain damage by inhibiting the JNK and p38 pathway.^44^ JNK‐IN‐8, a c‐Jun N‐terminal kinase inhibitor, improves functional recovery through suppressing neuroinflammation in ischemic stroke.^45^ In our study, these data demonstrated that IMP suppressed the phosphorylation of JNK, ERK, and p38 in LPS‐treated microglia.

As a key signal transduction factor in inflammatory response, NF‐κB plays a central role in inflammatory cytokine‐mediated inflammatory response.^46^ The activation of NF‐κB signaling aggravates the ischemic stroke injury, while inhibiting the phosphorylation of NF‐κB could reduce the brain damage.^47^, ^48^ Likewise, neural progenitor cell–derived extracellular vesicles reduces Evans blue extravasation and decreases ATP‐binding cassette transporter B1 expression by inhibiting the NF‐κB pathway in stroke mice.^49^ NF‐κB and MAPK signaling pathways promote nucleotide‐binding oligomerization domain (NOD)–like receptor (NLR) Pyrin domain containing 1 and 3 (NLRP1 and NLRP3) inflammasome activation in neurons following ischemic stroke.^50^ JZL184 and deletion of monoacylglycerol lipase inhibit the activation of the MAPK and NF‐κB pathways.^51^ However, further research is needed to determine whether IMP plays an important role in ischemic stroke through the MAPK and NF‐κB pathways.

In conclusion, our data uncovered that IMP exerted anti‐inflammatory effects in LPS‐induced primary microglia and attenuated ischemic stroke injury and neurological deficits in experimental stroke, which might be associated with the inhibition of MAPK and NF‐κB signaling pathways. These results suggested that IMP might provide an alternative treatment for ischemic stroke.

## CONFLICT OF INTEREST

Authors declare that there are no conflicts of interest associated with this article.

## Data Availability

The data that support the findings of this study are available from the corresponding author upon reasonable request.
